# An attempt to construct the individual model of daily facial skin temperature using variational autoencoder

**DOI:** 10.1007/s10015-021-00699-7

**Published:** 2021-09-24

**Authors:** Ayaka Masaki, Kent Nagumo, Yuki Iwashita, Kosuke Oiwa, Akio Nozawa

**Affiliations:** Aoyama Gakuin University, Tokyo, Japan

**Keywords:** Facial skin temperature, Infrared thermography, Deep learning, Anomaly detection, Variational autoencoder, Hotelling’s theory

## Abstract

Facial skin temperature (FST) has also gained prominence as an indicator for detecting anomalies such as fever due to the COVID-19. When FST is used for engineering applications, it is enough to be able to recognize normal. We are also focusing on research to detect some anomaly in FST. In a previous study, it was confirmed that abnormal and normal conditions could be separated based on FST by using a variational autoencoder (VAE), a deep generative model. However, the simulations so far have been a far cry from reality. In this study, normal FST with a diurnal variation component was defined as a normal state, and a model of normal FST in daily life was individually reconstructed using VAE. Using the constructed model, the anomaly detection performance was evaluated by applying the Hotelling theory. As a result, the area under the curve (AUC) value in ROC analysis was confirmed to be 0.89 to 1.00 in two subjects.

## Introduction

Facial skin temperature (FST) is an autonomic nervous system indicator and can be measured by infrared thermography. FST changes with physiological and psychological states and has been used in previous studies to estimate drowsiness [1], stress [2], and emotions [3]. However, there is an infinite number of states in which humans are physiologically and psychologically abnormal. For example, in some cases, it is enough to determine that a driver is awake when judging drowsiness or that a driver is healthy when performing a medical checkup. FST has also gained prominence as an indicator for detecting anomalies such as heat generation due to the COVID-19. When FST is used for engineering applications, it is enough to be able to recognize normal.

Masaki et al. confirmed that abnormal and normal conditions could be separated based on FST by using variational autoencoder(VAE) [4], a deep generative model [5]. Quantitative evaluation of anomaly detection performance has not yet been done. In the previous study, FST at rest measured temporarily was determined to be normal. However, it has been shown that FST fluctuates throughout the day according to circadian rhythms [6]. In other words, even if the body is normal, the FST is not constant, so the data from the temporary resting-state alone cannot reproduce the normal state of the FST.

The objective of this study is to construct the model of FST under normal conditions that takes into account diurnal variation. The reason for considering diurnal variation is to get closer to the model that can be used on a daily basis. In this study, normal FST with a diurnal variation component was defined as a normal state, and a model of normal FST in daily life was individually reconstructed using VAE. The normal data used in the experiment was the FST when the diurnal variation was encouraged by recording once an hour from 8:00 to 23:00 in a day. The data under abnormal conditions, such as simulated fever, is the same as in the previous study when the FST was subjected to extreme changes by holding one’s breath. After attempting to construct a model of daily FST by VAE, the anomaly detection performance was evaluated for each subject using the Hotelling theory [8], a statistical threshold determination method.

## Variational autoencoder

### Overview of the VAE algorithm

**Fig. 1:**
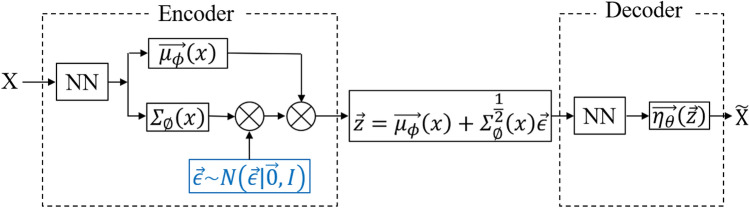
Overview of VAE

VAE is a generative model based on deep learning. Figure 1 shows an overview of the VAE algorithm. X, $\widetilde{X}$, NN represent input, output, and a neural network, respectively. The VAE network is divided into an encoder section and a decoder section. Given observation $X = \{\overrightarrow{x_1}, \overrightarrow{x_2},..., \overrightarrow{x_N}\}$, VAE identify probability distributions ($p(\overrightarrow{x_*}|X)$) that produce unobserved value ($\overrightarrow{x_*}$). VAE is designed on the assumption that latent variables that serve as explanatory variables are normally distributed. The encoder performs dimensional compression of X and it calculates the mean vector ($\overrightarrow{\mu _{\phi }}\left( x\right)$) and variance ($\Sigma _{\phi }\left( x\right)$), which are parameters of the normally distribution. The blue part in Fig. 1 indicates that sampling is performed from the standard normal distribution. The points are then sampled from the latent space distribution($\overrightarrow{z}$). In the decoder, the model likelihood parameters ($\overrightarrow{\eta _{\theta }}\left( z\right)$) is calculated, and the reconstruction error can be computed. Finally, the reconstruction error is backpropagated through the network. Since the reconstruction error to be optimized includes a regularization term that brings the mean ($\overrightarrow{\mu _{\phi }}\left( x\right)$) to 0 and the variance ($\Sigma _{\phi }\left( x\right)$) close to the unit matrix, the distribution of the latent variable $\overrightarrow{z}$ has a shape close to a standard normal distribution. There is a tendency to regularize the organization of the latent space by bringing the distribution returned by the encoder closer to the standard normal distribution. For this reason, VAE can avoid overfitting and achieve a high recall compared to a normal autoencoder.

### Construction of the model of daily facial skin temperature for anomaly detection using VAE

We explain the concept of anomaly detection in FTIs using VAE. Normally, with machine learning, it is necessary to have a dataset with a complete number of samples for each class. However, it is difficult to collect data when a person is abnormal (e.g., data on people who are not feeling well). Therefore, in this study, an evaluation of the model constructed using only the data in the normal condition, which is relatively easy to collect, was performed. In this study, we defined facial thermal image (FTI) in a healthy state during daily life as Normal, in unusual cases as Anomaly. Various Normal is used for anomaly detection using VAE. Firstly, by learning a large amount of Normal using VAE, the model of daily FST was constructed. Secondly, testing data (Anomaly and Normal) were input to the model of daily FST. If the pattern was similar to Normal, the testing data was decided Normal; otherwise, the testing data was decided Anomaly.

## Experiment

### Experiment system

The experimental systems consisted of an infrared thermography device (FLIR A35-Series, FLIR systems Co., Ltd). The infrared thermography device was placed at 1.0 m in front of the subject. The size of the thermal image was 320 $\times$ 256 pixels, and the temperature resolution was less than $0.05\,^\circ$C. The sampling frequency of thermal images was 1 Hz. The infrared emissivity of skin was $\varepsilon$ = 0.98.

### Procedure and condition

**Fig. 2:**
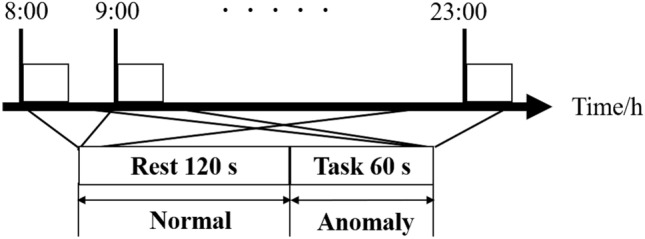
Experimental protocol

The subjects were two 22- to 24-year-old men. The subjects provided informed consent about the experiments and objects of this study prior to agreeing to participate in the experiment. The experiments were conducted in an experimental room (Average room temperature: 22.1$^\circ$C). The subjects have entered the room 15 minutes before the beginning experiment to acclimate the room temperature. The measurement was conducted at every hour, 8:00 to 23:00 for a day. To control physiological responses to eating, subjects were inhibited from eating between meals except for breakfast, lunch, and dinner. Figure 2 shows the experimental protocol. The experiment consists of resting-state segments (Rest) and an inducing acute-stress physiological response segment (Task). In the Rest, subjects were resting with their eyes closed for 120 s. In the Task, subjects were asked to hold their breath with their eyes closed for 60 s to change FST forcibly.

## Analysis methods

### Definition of normal and anomaly

**Fig. 3:**
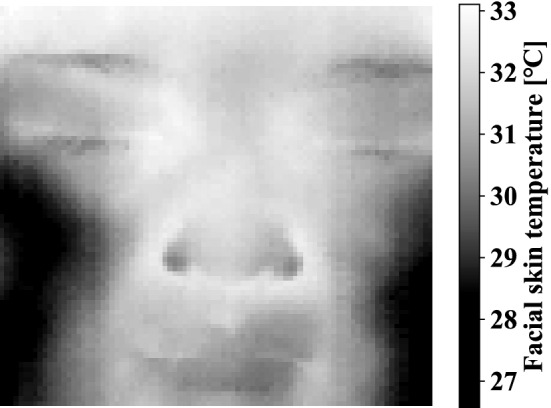
Sample of facial thermal image

To construct the model of daily FST using VAE requires a large amount of normal data. One of the differences from previous study [5] is the method of obtaining normal data. In this study, the Normal was measured at every hour, from 8:00 to 23:00, for a day to take into account the human circadian rhythm. Anomaly is FTIs in which the skin temperature is forcibly changed by holding the breath to simulate abnormalities such as fever. Image sizes are 320 $\times$ 256 pixels. The number of normal data was 1920 (= 120 $\times$ 16), of which 90% was used as learning data, and any 60 out of 10% was used as testing data. The Anomaly for evaluation were samples of the last 20 s of a 1-min Task at any time. The difference in the FST value in a single image is small. The thermal images may also be biased by room temperature and other disturbances. In this study, the FTI was normalized such that the maximum temperature was 1 and the minimum temperature was 0 for learning the relative amount of skin temperature inside the face. Specifically, the 320 $\times$ 256 pixels thermal image output from the thermographic device was cropped so that the subject’s face remained. This thermal image after cropping is defined as a FTI. FTI size depends on the size and shape of the subject’s face. FTI sizes of subA and subB were actually 90 $\times$ 83 and 73 $\times$ 94 pixels, respectively. An example of an FTI is shown in Fig. 3. The normalization was applied independently to each FTI.

**Fig. 4:**
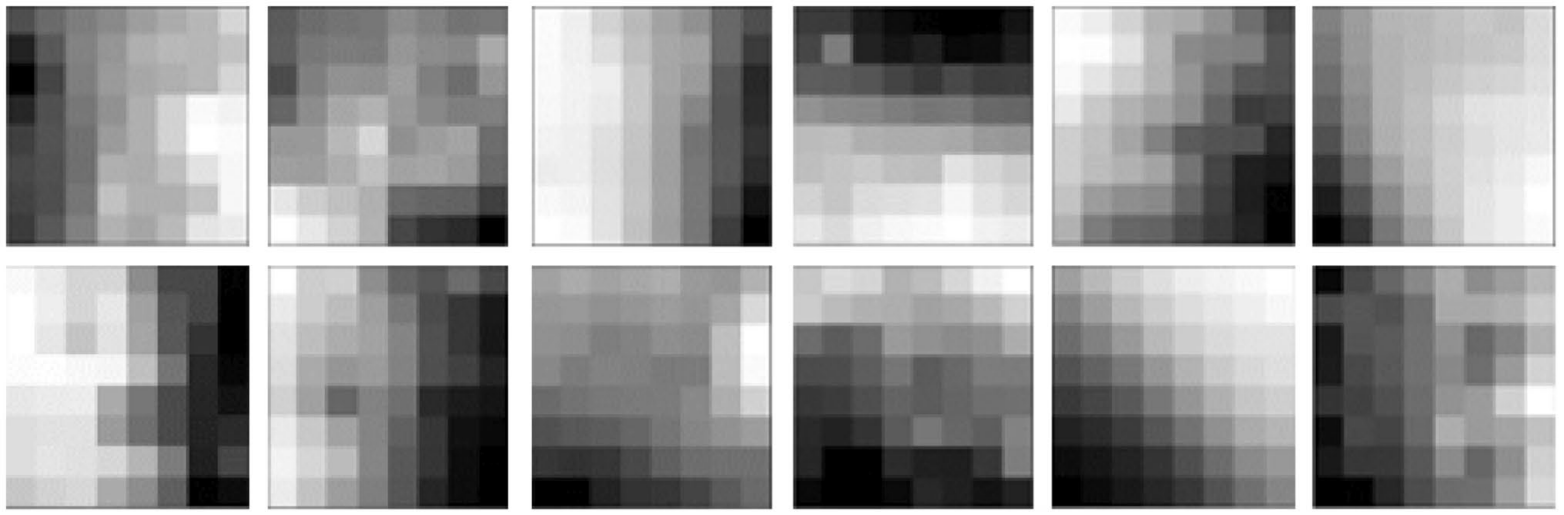
Samples of learning data

### The model construction

The method of constructing the model is almost the same as in previous studies [5]. In this study, an individual model was constructed for each subject. When performing VAE learning, a part of the FTI is randomly cut out at 8 $\times$ 8 size pixels, and this patch is used to learn the fine vascular pattern of skin temperature as learning data as shown Fig. 4. The learning data for subA and subB have been expanded to 10,000 sheets and 50,000 sheets, respectively. The expansion method follows the previous study [5]. In this study, convolutional layers were placed before the FC layer of the encoder in order to extract the features of the skin temperature pattern of the skin blood vessels. Along with that, deconvolutinal layers were placed after the decoder. The construction of the encoder is depicted in Table 1. The structure of the VAE encoder consisted of two convolutional layers and one fully connected layer. In this table, Conv, BatchNorm, and FC indicate the convolutional, batch normalization, and fully connected layers, respectively. The mean vector and variance were output from FC. The structure of the decoder is paired with the structure of the encoder, and the structure is opposite to that of the encoder. The construction of the decoder is depicted in Table 2. ConvTrans indicates a transpose convolution. The gradient descent method was used for VAE parameter learning, and the optimization algorithm at that time was Adam. The number of dimensions of the latent variable $\overrightarrow{z}$ was 6, and the best model was selected. The number of epochs was 15, and the batch size was 128.

When testing, for all test data, the spatial unregularized anomaly score was calculated with reference to [7] and used as an index for detecting FTIs with some abnormality from multiple FTIs. Unregularized anomaly score represented the error between the input vector and the reconstructed vector. To use the unregularized anomaly score to determine Anomaly, it is necessary to set a threshold mostly. Determining the threshold for the next subsection is the purpose of this study. The equation for the spatial unregularized anomaly score is shown below.

1 $$\begin{aligned} L_{VAE}\left( x\right) = \left. \sum ^{Nx}_{i=1}\frac{1}{2}\times \frac{\left( \mu _{xi}-x_{i}\right) ^{2}}{\sigma ^{2}x_{i}} \right| _{z=\mu _{z}}. \end{aligned}$$

The above equation is directly related to the reproduction error. *Nx* and $x_{i}$ represent the number of pixels in the image and any pixel value, respectively. $\mu _{xi}$ represents the maximum posterior probability estimation of the latent variable $\overrightarrow{z}$. The previous study suggests that the mean and variance of this score separates Normal from Anomaly [5]. In this study, these statistics were treated as a sample, and statistical methods were used for anomaly detection.

**Table 1:** Construction of encoder

Layers	Function	Filters	Size	Stride
Conv1	–	16	2$\times$2	2
BatchNorm1	ReLU	–	–	–
Conv2	–	32	2$\times$2	2
BatchNorm2	ReLU	–	–	–
FC	–	–	–	–

**Table 2:** Construction of decoder

Layers	Function	Filters	Size	Stride
FC	–	–	–	–
BatchNorm1	ReLU	–	–	–
ConvTrans1	–	32	2$\times$2	2
BatchNorm2	ReLU	–	–	–
ConvTrans2	–	16	2$\times$2	2
BatchNorm3	ReLU	–	–	–
ConvTrans3	–	1	4$\times$4	1
ConvTrans4	–	1	4$\times$4	1
BatchNorm4	ReLU	–	–	–
BatchNorm5	ReLU	–	–	–

### Model evaluation methods for anomaly detection

In this study, anomaly detection was performed using an unregularized anomaly score calculated from the model learned local image of daily FST and testing data. The evaluation method used was the Hotelling theory, parametric methods. In this study, the Shapiro-Wilk test confirmed the normality of the statistic of unregularized anomaly score. Since Unregularized anomaly scores tend to diverge, they were converted to a logarithmic scale. The testing data consists of 60 Normal samples that are not used for learning the model and 20 Anomaly samples from the last 20 s of the Task. Thus, the total sample size is 80. The results of the Shapiro–Wilk test showed that the *p*-values of the mean and variance of the unregularized anomaly score were both greater than 0.05, confirming that the distribution was parametric. Therefore, the Hotelling theory, one of the parametric anomaly detection methods, was adopted in this study.

The following shows the method of applying the hoteling theory in this study. $X'$ represents the mean and variance of the unregularized anomaly score. Also, $x'$ represents an element of $X'$.

1. An average $\hat{\mu }$ and variance $\Sigma$ are calculated by maximum likelihood estimation.
2. The following squared value of Mahalanobis distance $a_{(x')}$ (2) were calculated for all data $x'$. $a_{(x')}$ represents the degree of anomaly. It can be approximated by $\chi ^{2}$ distribution with 1 degree of freedom.
  2 $$\begin{aligned} {a_{(x')} = (\mathbf{x'} - {\hat{\mu }})^\mathrm{T} \Sigma ^{-1} (\mathbf{x'} - {\hat{\mu }}) } \end{aligned}$$
3. For each data, the following equation (3) was used as a measure of anomaly detection. is calculated for *x*. *P* stands for Probability of occurrence of anomaly samples.
  3 $$\begin{aligned} P=\int ^{\infty }_{\alpha _{x}}\chi ^{2}du \end{aligned}$$
4. Back-calculate the anomaly threshold $a_{(x')}$ from the desired anomaly detection probability.
5. If the anomaly threshold is greater than the threshold, it is judged as Anomaly.

## Results and discussion

**Fig. 5:**
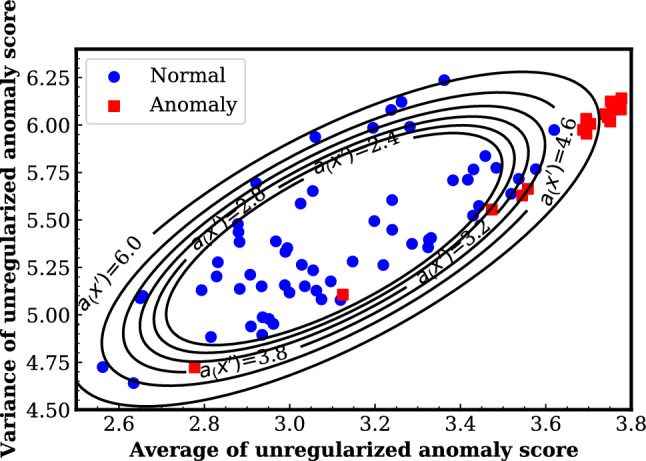
Probability of being detected as abnormal and range of normal data. The contour lines correspond to the cases where 30%, 25%, 20%, 15%, 10%, and 5% of the samples from the inside are detected as anomalies (subA)

**Fig. 6:**
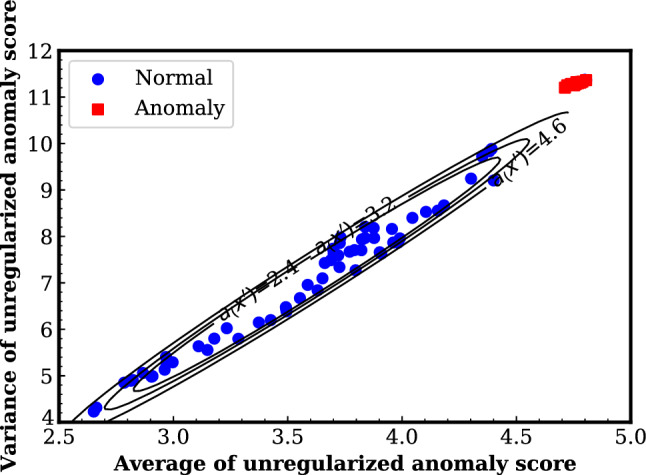
Probability of being detected as abnormal and range of normal data. The contour lines correspond to the cases where 30%, 20%, and 10% of the samples from the inside are detected as anomalies (subB)

Figures 5 and 6 show the result of visualizing the anomaly threshold as a contour plot. The vertical and horizontal axes are the variance and mean of the unregularized anomaly score, respectively. The contour lines of Fig. 5 represents the cases where the probability of an anomaly being detected is set to 30%, 25%, 20%, 15%, 10%, and 5%, respectively, from the inside. $a_{(x')}$ is the squared value of the Mahalanobis distance. Figure 6 is almost the same appearance as Fig. 5, but the number of contour lines has been reduced to make it easier to read. In this study, the value of area under the curve (AUC) in ROC analysis was used as an indicator. The AUC of subA and subB were 0.89 and 1.00, respectively. As shown in the results, in the case of subA, the identification accuracy changes depending on the threshold, while in the case of subB, identification is possible at any threshold. This can be attributed to two things: the first is that the learning of local information of FST by VAE has individual differences, and the second is that the threshold needs to be determined for each individual. In addition, it is difficult to discriminate between normal and abnormal with 100% probability using our method. However, we believe that there are situations where our method can be applied by setting the probability of detecting abnormality as needed. Another limiting factor of this research is the low stability of learning due to a lack of data. In the future, it is necessary to increase the number of data and subjects to reduce the stability of learning.

## Conclusion

In this study, normal FST with a diurnal variation component was defined as a normal state, and a model of normal FST in daily life was individually reconstructed using VAE. Based also on the statistics of the unregularized anomaly score calculated from the constructed model, the anomaly detection performance was evaluated by applying the Hotelling theory. As a result, the AUC value in ROC analysis was confirmed to be 0.89 to 1.00 in two subjects.
